# Utilization of tissue-free minimal residual disease testing in colorectal cancer patients from Asia and Middle East

**DOI:** 10.3389/fonc.2024.1426941

**Published:** 2024-09-20

**Authors:** Suyog Jain, Shaheenah Dawood, Viraj Lavingia, Dan Aderka, Esther Tahover, Yao-Yu Hsieh, Mark Temper, Alesya Goldman, Marwan AI. Akasheh, Steve Olsen, Sandra San Hsing, Nisarg Joshi, Hsiao-Yu Jen

**Affiliations:** ^1^ Medical Affairs Department, Guardant Health Pte Ltd, Singapore, Singapore; ^2^ Department of Medical Oncology, Mediclinic City Hospital, Mohammed Bin Rashid University of Medicine and Health Sciences, Dubai, United Arab Emirates; ^3^ Department of Medical Oncology, Shalby Hospital, Ahmedabad, India; ^4^ Oncology Department, Assuta Ramat Hahayal, Tel Aviv-Yafo, Israel; ^5^ Division of Hematology and Oncology, Shuang Ho Hospital, Taipei Medical University, New Taipei City, Taiwan; ^6^ Division of Hematology and Oncology, Department of Internal Medicine, School of Medicine, College of Medicine, Taipei Medical University, Taipei, Taiwan; ^7^ Center of Oncology, Hadassah University Medical Center, Jerusalem, Israel; ^8^ Oncology Department, Meir Medical Center, Kfar-Saba, Israel; ^9^ Department of Oncology, Maggi Medical Centre, Amman, Jordan

**Keywords:** MRD - molecular residual disease, CRC - colorectal cancer, ctDNA - circulating tumour DNA, liquid biopsy, Reveal, epigenomics, methylation

## Abstract

**Introduction:**

The presence of minimal residual disease (MRD) after curative-intent surgery for early-stage cancers is associated with disease recurrence. Circulating tumour deoxyribonucleic acid (ctDNA) has emerged as a promising biomarker for MRD assessment in patients with colorectal cancer (CRC) who have undergone surgery or completed adjuvant therapy. MRD tests are already available for use in clinics; however, treatment decisions following MRD results obtained in routine practice are infrequently described.

**Methods:**

In this observational study, we report on the real-world clinical use of Guardant Reveal, a validated tissue-free MRD assay, in the first 215 consecutive patients (279 samples) with CRC tested in Asia and the Middle East.

**Results:**

Overall, 22% of patients had ctDNA detected in their first MRD test, and the frequency of ctDNA positivity increased with increasing tumour stage. 132 samples were tested with an earlier version of Guardant Reveal, one that assessed both genomic and epigenomic features. An updated version of the assay assesses only ctDNA methylation data and was used for the remaining 147 samples. In patients with stage II CRC, 71% of tests were ordered within 12 weeks after tumour resection, while for patients with stage III disease, 69% of tests were ordered after completion of all curative-intent treatment.

**Discussion:**

Clinical cases utilizing tissue-free MRD assessment are described.

## 1 Introduction

In 2018, among all genders and age groups, Asia had the highest proportions of both incidence (51.8%) and mortality (52.4%) for colorectal cancer (CRC) of any region in the world (1). In particular, a higher incidence of CRC has been observed in more economically developed countries such as Japan, South Korea, Singapore, and Taiwan (1). Approximately 75% of newly diagnosed CRC patients present with non-metastatic early-stage disease, which presents an opportunity for curative-intent treatment (2). Despite surgery and adjuvant therapy for CRC, as many as 33% of patients experience recurrent disease (3). Therefore, improvements in the diagnosis and treatment of early-stage CRC remain an important unmet need.

Typically, clinicopathologic features are assessed to determine the prognosis of a patient after surgery for early-stage CRC. Such features that have been associated with a worse prognosis in CRC patients are a T4 primary; high-grade/poorly differentiated histology; lymphovascular invasion; perineural invasion; clinical bowel obstruction or perforation; close, indeterminate, or positive margins; inadequately sampled lymph nodes; a high preoperative serum carcinoembryonic antigen (CEA) level; and high levels of tumour budding. Based on these factors, patients at higher risk are generally offered adjuvant chemotherapy. However, the accuracy of clinicopathological criteria alone to identify patients at risk for recurrence is being challenged by emerging evidence (4, 5).

Minimal residual disease (MRD) refers to the presence of cancer cells that are below detectable levels with conventional diagnostic methods. These cells persist in the body after the completion of definitive therapy (surgery with or without adjuvant chemotherapy) (4) and are often the cause of cancer recurrence (6, 7).

Circulating tumour DNA (ctDNA), tumour-derived single- or double-stranded DNA fragments detectable in the plasma, has emerged as a promising biomarker for MRD assessment in patients with CRC who have undergone curative-intent surgery or completed adjuvant therapy (8). Several prospective studies have reported on the role of ctDNA for the prognosis of disease recurrence after the completion of definitive treatment (9). These studies suggest that ctDNA-based MRD assessment may outperform existing clinicopathologic criteria-based risk-stratification strategies. Prospective clinical trials are investigating the impact on patient outcomes after intervening based on MRD status, such as PEGASUS (NCT04259944), VEGA (jRCT1031200006), IMPROVE-IT2 (NCT04084249), and ACT-3 (NCT03803553).

MRD assessment can be performed with or without tumour tissue from surgery. Tissue-informed assays require prior knowledge of the tumour genomic profile for each patient, generally acquired by whole-exome sequencing or targeted sequencing of the primary tumour (e.g., Signatera™, SafeSeqS) (10). These assays are personalized to detect patient-specific genomic alterations via targeted sequencing of plasma cell-free DNA. On the other hand, a tissue-free assay utilizes broad panel-based next-generation sequencing without prior knowledge of the patient’s tumour mutational profile. For example, Guardant Reveal™ was originally designed to detect both genomic alterations and differential DNA methylation signatures known to occur in a given tumour type (9). Recently, the assay has been modified to focus only on identifying tumour-derived methylation signatures (11). Early versions of this assay demonstrated a sensitivity of 50% when measured at a single time point after the completion of curative-intent treatment, increasing to 91% with serial sampling (12). The current version of the assay reported 81% sensitivity with serial samples for colon cancer, and post treatment sample level specificity of 98.2% (11). The average lead time from MRD detection to radiographic confirmation of disease recurrence was 200 days (12).

ctDNA-based MRD tests are already available for use in clinics, but how physicians use the results in real-life settings has been infrequently described. In this analysis, we describe the real-world clinical use of a validated tissue-free MRD assay (Guardant Reveal) in consecutive patients with early-stage colorectal cancer from Asia and the Middle East. We also describe a selected subset of clinically annotated cases as representative of the utility of the MRD assay results for making treatment decisions.

## 2 Materials and methods

Guardant Reveal (Guardant Health, Inc) is a tissue-free MRD test for the detection of cancer. An earlier version of this commercially available panel was 500kb (50kb genomic and 450kb epigenomic), while the current version is 15 Mb and utilizes the expanded genomic footprint to identify a broader range of tumour-derived methylation signatures (**Supplementary Table 1**) (11). This MRD test can be ordered for stage I, stage II, stage III and oligo metastatic stage IV. The analytical details have been previously described (11). The first 215 consecutive early-stage CRC patients with 279 longitudinal samples from Asia and the Middle East with test results from commercially available versions of Guardant Reveal were queried retrospectively. The first 108 consecutive patients’ samples (n=132) collected between November 2021 to June 2023 were tested on the 500 kb panel, and, since July 2023, samples (n=147) were tested on the 15 Mb panel. Findings were analysed through the data cut-off of 20 Feb 2024 for patients who had a test result and documented clinical stage. Patients could have been tested post-resection (within 12 weeks of surgery) or during surveillance (more than 12 weeks after surgery). Clinical factors (age, sex, stage at diagnosis, date of surgery, adjuvant therapy added or not, timing of MRD test ordered) were extracted from test requisition forms submitted when the test was ordered. Additional information from selected patients was included to describe the actions taken by treating physicians based on the test results. Individual patients’ consent to use deidentified data was obtained by their respective treating physicians.

## 3 Results

### 3.1 Demographics

A total of 215 patients (279 samples) had an MRD test result with cancer stage documented. Among these, 108 patients were assessed with the early version of Guardant Reveal, and 107 patients were assessed with the currently available version. Demographics details are shown in **Table 1**.

**Table 1:** Demographics.

Patient Characteristic	No. (n=215) (%)
Gender	
Male	138 (64%)
Female	77 (36%)
**Median Age (range)**	59 (17-90 yrs)
Cancer Stage	
I	0 (0%)
II	82 (38%)
III	122 (57%)
IV (oligo metastatic)	11 (5%)
Region	
Taiwan	50 (23%)
Israel	47 (22%)
India	33 (15%)
Middle East	30 (14%)
Other Asian countries	55 (26%)

### 3.2 Test utilization and ctDNA positivity by cancer stage

**Figure 1:**
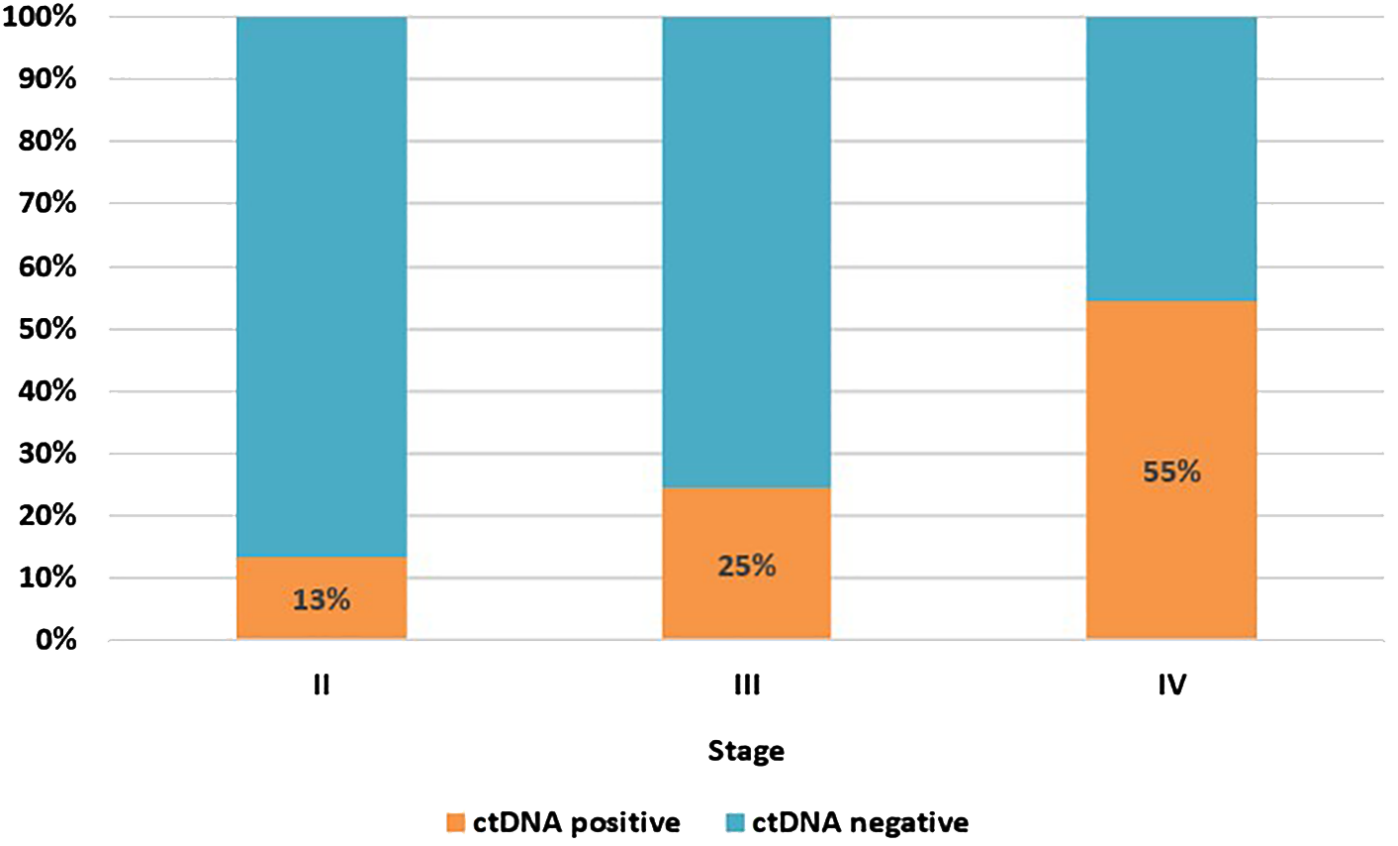
ctDNA positivity rate at first MRD test by cancer stage. Stage II, n=82; stage III, n=122; stage IV (oligo metastatic), n=11.

As some of the patients had more than one MRD test, the calculation of ctDNA positivity was based on the result of the first MRD test. Overall, 22% of patients had ctDNA detected in their first MRD test, 19% for those conducted post-resection and 26% for first tests during surveillance; 22% of all samples were positive, regardless of timing. Based on the result of the first test alone, the proportion of patients with ctDNA detection increased with increasing cancer stage, stage II 13%; stage III 25%; oligo metastatic stage IV, 55% (**Figure 1**).

**Figure 2:**
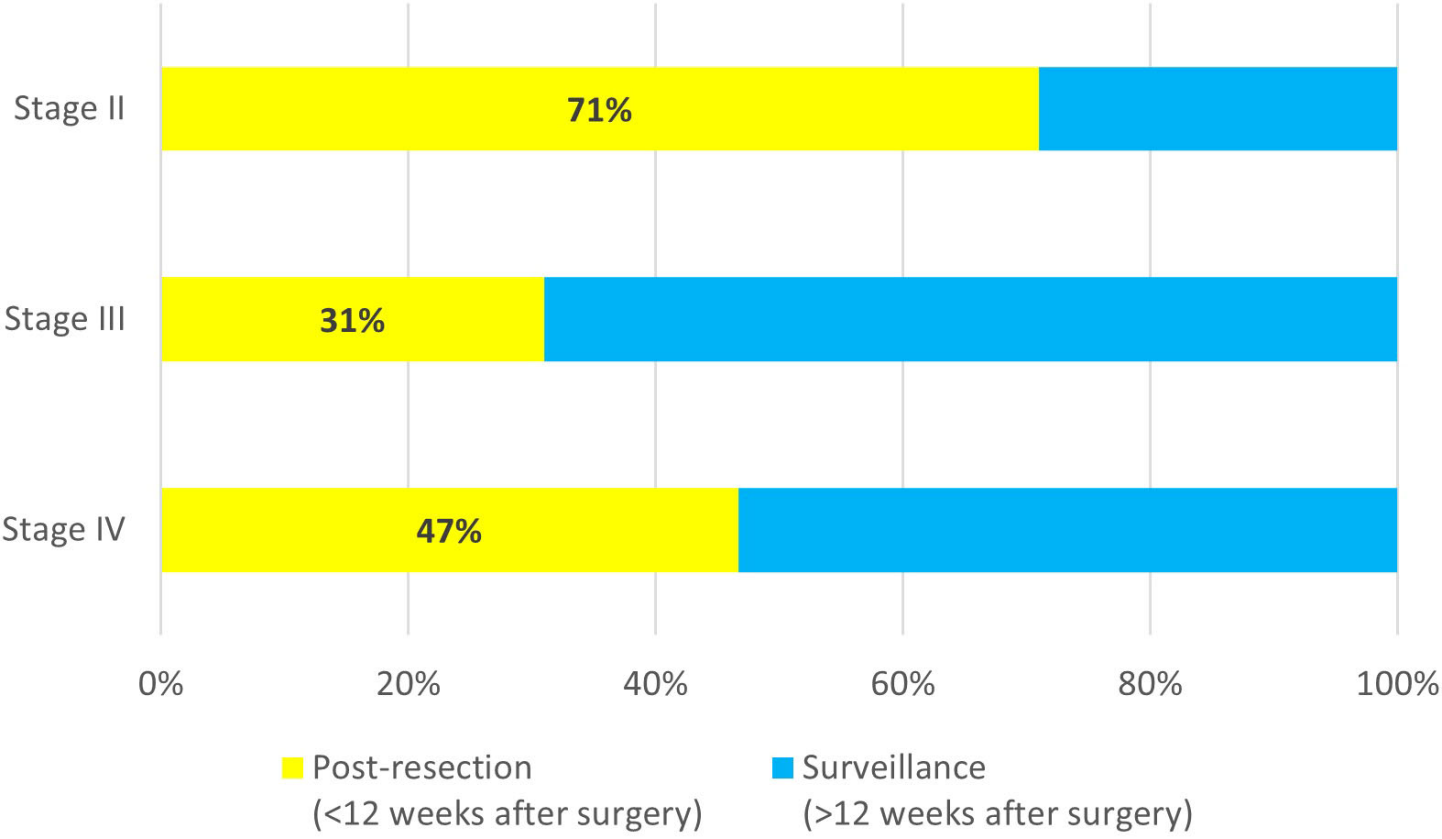
Timing of MRD test order for stage II (n=93), stage III (n=171), and oligo metastatic stage IV (n=15).

Among all tests for patients with stage II disease, 71% (66/93) were ordered within the first 12 weeks after surgery. For patients with stage III disease, the majority of tests were ordered during surveillance (118/171, 69%) (**Figure 2**). The median time for ordering a post-resection MRD test was 6 weeks (range 2 to 12 weeks) after surgery for both stage II and stage III patients, while the median time for ordering the first surveillance MRD test was 21 weeks (range 13 to 606 weeks) after resection for stage II patients, and 39 weeks (range 13 to 737 weeks) after resection for stage III patients. For patients with oligo metastatic stage IV disease, 47% (7/15) were ordered within the first 12 weeks after surgery. The median time for ordering a post-resection MRD test was 5 weeks (range 3 to 11 weeks) after surgery, and the median time for ordering the first surveillance MRD test was 34 weeks (range 22 to 108 weeks) after resection.

Among the 61 ctDNA positive samples, 32 were reported on the earlier commercial version of Guardant Reveal (52%) and 29 on the currently available version of assay (48%).

Although the updated version of the assay detects MRD based solely on methylation signals, MRD detection rates (21%) were similar to the earlier version (23%) which assessed mutations and methylation.

The median turnaround time (TAT) from sample reaching the laboratory until report release was 10 days (range: 5-29 days) for the earlier commercial version of the assay and 9 days (range: 5-26 days) for the currently available version.

### 3.3 Selected cases using MRD results for treatment decisions

Selected patients whose detailed treatment history before and after MRD test results were available are shown in **Table 2** and **Table 3**. **Table 2** includes cases for which a single MRD test was ordered while **Table 3** includes cases with longitudinal testing (2 or more tests for the same patient). All the patients in **Table 2** and **Table 3** used the earlier commercial version of the assay.

**Table 2:** Clinical factors and impact of a single MRD test result on clinical decision making.

Case	CRC Stage	Region	Treatment plan before ordering MRD test	Timing of MRD test order	MRD test result	Treatment plan after the MRD test results	Impact of the MRD test result
1	II colon cancer	Israel	The patient declined adjuvant therapy despite the physician’s recommendation.	4 weeks after surgery	Positive	Adjuvant chemotherapy	MRD test result persuaded the patient to initiate adjuvant treatment.
2	III colon cancer	Taiwan	Adjuvant chemotherapy x 12 cycles	4 weeks after surgery	Positive	Maintained original treatment plan	MRD test results improved confidence in the original treatment plan.
3	III colon cancer	Jordan	Adjuvant chemotherapy x 6 cycles	4 weeks after surgery	Positive	Maintained original treatment plan	MRD test results improved confidence in the original treatment plan.
4	IV colon cancer Oligo-metastatic	Israel	Observation	4 weeks after surgery	Positive	Imaging and adjuvant chemotherapy	MRD test result led to the initiation of chemotherapy.
5	III rectal carcinoma	India	Neoadjuvant therapy followed by surgery then close observation	4 weeks after surgery	Positive	Adjuvant chemotherapy	The treatment plan changed based on surgical findings and evidence of MRD.
6	IV rectal carcinoma Oligo-metastatic	India	Adjuvant chemotherapy	After completion of adjuvant chemotherapy	Positive	Intensified chemotherapy to include irinotecan	The chemotherapy regimen intensified based on the MRD result.
7	IV sigmoid carcinoma Oligo-metastatic	UAE	Adjuvant chemotherapy	4 weeks after surgery	Negative	No adjuvant chemotherapy; observation	Negative MRD results supported observation only.
8	II colon cancer	Israel	Observation	4 weeks after surgery	Negative	Maintained original treatment plan	Negative MRD result supported observation.

**Table 3:** Clinical factors and utility of longitudinal MRD tests for individual patients.

Case	CRC Stage	Region	Treatment plan before ordering the first MRD test	Timing and result of first MRD test result	Treatment plan after the first MRD test result	Timing and result of the second MRD test result	Treatment plan after the second MRD test result	Impact of the MRD test result
10	II	Israel	Observation, but ambiguous radiographic finding 12 weeks after surgery	12 weeks after surgery Positive	Curative intent resection of the recurrent lesion	4 weeks after resection of recurrent lesion Positive	Administer chemotherapy	MRD result confirmed suspected recurrence, leading to further surgery and adjuvant therapy.
11	III	Israel	Adjuvant chemotherapy x 3 months	After 3 months chemotherapy Positive	Continue chemotherapy for 3 more months (6 months total)	After 6 months chemotherapy Positive	Follow-up with imaging every 3 months	MRD positive after 3 months chemotherapy supported decision to continue treatment.
12	III	UAE	Chemotherapy x 6 cycles; patient wanted to stop after 2 cycles due to adverse events	After 2 cycles chemotherapy Positive	Patient continued chemotherapy to complete 6 cycles	After 6 cycles chemotherapy Negative	Standard follow-up	MRD result encouraged patient to complete chemotherapy.
13	II	India	Adjuvant chemotherapy for 3 months. However, after 4 cycles of adjuvant chemotherapy, patient wished to stop due to severe adverse event.	17 weeks after surgery Positive	Changed to another chemotherapy for 3 months	After 6 cycles FOLFIRI treatment Negative	Standard follow-up Disease free for 18 months	MRD result encouraged patient to complete chemotherapy.

## 4 Discussion

Emerging data suggest that the presence of ctDNA after resection of early-stage CRC is prognostic for recurrence (9). There is evidence that ctDNA-guided risk stratification in patients with resected CRC may even outperform established methods that are based on clinicopathologic features (9). Recently published data from COSMOS-CRC01 study on current version (11) indicated similar performance to the early version of the Reveal test (12). Even as the number of clinical trials of MRD is increasing, limited data have been reported regarding real-world use of such tests. Therefore, we have described the initial experience with a commercially available tissue-free MRD assay in Asian and Middle Eastern patients with resectable colorectal cancer.

Among the patients tested, the ctDNA positivity rate based on the result of the first MRD test was 22%, similar to that observed in a study of the same assay in predominantly Caucasian patients (13). As the assay has evolved, and considering potential differences in patient and their tumour characteristics between testing cohorts, the positive detection rate was similar with the earlier version and the current version. We have not attempted to make any comparisons between previous and current version of assay in this cohort as the patient characteristics cannot be controlled in the real-world setting.

We also observed that among stage II colorectal cancer patients, 71% of all tests were ordered within 12 weeks of surgery, presumably to support adjuvant therapy decision-making. In contrast, for patients with stage III CRC, most MRD tests were conducted during or after completion of adjuvant therapy (69%), in part because adjuvant chemotherapy is standard care in this setting but also because the optimal duration of adjuvant treatment remains unclear (14).

Because timely adjuvant treatment within 8-12 weeks of resection is essential (15), a clinically useful turnaround time for any MRD test is necessary. Based on our experience, the tissue-free MRD test can be ordered 4 weeks after resection and physicians can receive the results (median TAT of 9 days) within the recommended window for initiation of adjuvant chemotherapy. This TAT is similar to that reported for a similar cohort of patients in the United States using the same test (13).

Practicing physicians among the authors opine that, despite the absence of robust guidelines regarding the use of MRD test findings, physicians in clinical practice are already making treatment decisions that consider the results of MRD testing. We observed that, following a positive MRD report, physicians tended to escalate treatment. Such approaches included initiating chemotherapy in patients not originally considered for it, prolonging the duration of previously planned chemotherapy, or changing the chemotherapy regimen. After a negative MRD report physicians chose to follow the patient as per the local standard of care. The laboratory does not provide therapeutic recommendations in its reports. Therefore, all treatment decisions were made independently by the ordering physicians in collaboration with their patients.

There are several limitations to our report. The relatively small cohort size of 215 patients does not necessarily represent an unselected CRC patient population. It is likely that the population is enriched for cases involving exceptional scenarios for which MRD testing was employed when clinical findings and patients’ preferences diverged from standard treatment guidelines. With the data reported thus far for the MRD test, positive predictive value (PPV) has been robust and considered more reliable to change treatment decisions compared to negative predictive value (NPV). Therefore, it is possible that there was a preference for altering the treatment plan after a positive report compared to a negative report. All the MRD tests were ordered in a real-world scenario; therefore, there is no standardization of duration or regimen of chemotherapy as well as the timing of ordering the MRD test. Treatment details of all 215 patients are not available, therefore only selected patients are included in **Tables 2**, **3**. However, cases where MRD results changed or did not change treatment plans were observed. For most patients, follow-up duration is short, prohibiting assessment of the specificity and sensitivity of the MRD assay in predicting recurrences. Similarly, due to the short duration of follow-up, we cannot estimate the impact of interventions on disease-free and overall survival.

Important clinical questions on the use of MRD tests for patients with early-stage colorectal cancer are being addressed in ongoing prospective studies (PEGASUS, VEGA, ALTAIR, IMPROVE-IT2, ACT-3). While awaiting results from these studies, physicians are already using MRD test results to complement established clinical criteria to inform treatment decisions for their patients with early-stage colorectal cancer. As both tissue-informed and tissue-free assays are being assessed across several prospective studies, we await further validation of the potential clinical advantages and limitations of this emerging technology.

## 5 Conclusion

Through retrospective analysis of real-world data, we describe how physicians in Asia and the Middle East currently apply a commercially available tissue-free MRD assay in clinical decision-making for patients after surgical resection of colorectal cancer.

Results from prospective randomized studies will further define the clinical role of MRD detection in resectable CRC.

## Data Availability

The original contributions presented in the study are included in the article/**Supplementary Material**. Further inquiries can be directed to the corresponding author.

## References

[1] Onyoh EF Hsu W-F Chang LC Lee YC Wu M-S Chiu H-M. The rise of colorectal cancer in Asia: epidemiology, screening, and management. Curr Gastroenterol Rep. (2019) 21:36. doi: 10.1007/s11894-019-0703-8 31289917

[2] Chakrabarti S Peterson CY Sriram D Mahipal A. Early stage colon cancer: Current treatment standards, evolving paradigms, and future directions. World J Gastrointest Oncol. (2020) 12:808–32. doi: 10.4251/wjgo.v12.i8.808 PMC7443846 32879661

[3] Guraya SY. Pattern, stage, and time of recurrent colorectal cancer after curative surgery. Clin Colorectal Cancer. (2019) 18:e223–8. doi: 10.1016/j.clcc.2019.01.003 30792036

[4] Luskin MR Murakami MA Manalis SR Weinstock DM. Targeting minimal residual disease: a path to cure? Nat Rev Cancer. (2018) 18:255–63. doi: 10.1038/nrc.2017.125 PMC6398166 29376520

[5] Badia-Ramentol J Linares J Gómez-Llonin A Calon A. Minimal residual disease, metastasis and immunity. Biomolecules. (2021) 11:130. doi: 10.3390/biom11020130 33498251 PMC7909268

[6] Jessup JM Goldberg RM Aware EA Benson A Brierley J Chang G. Colon and rectum. In: AJCC cancer staging manual, 8th. AJCC, Chicago (2017). p. 251.

[7] Osterman E Glimelius B. Recurrence risk after up-to-date colon cancer staging, surgery, and pathology: analysis of the entire swedish population. Dis Colon Rectum. (2018) 61:1016–25. doi: 10.1097/DCR.0000000000001158 30086050

[8] Chakrabarti S Xie H Urrutia R Mahipal A. The promise of circulating tumor DNA (ctDNA) in the management of early-stage colon cancer: A critical review. Cancers (Basel). (2020) 12:2808. doi: 10.3390/cancers12102808 33003583 PMC7601010

[9] Parikh AR Van Seventer EE Siravegna G Hartwig AV Jaimovich A He Y. Minimal residual disease detection using a plasma-only circulating tumor DNA assay in patients with colorectal cancer. Clin Cancer Res. (2021) 27:5586–94. doi: 10.1158/1078-0432.CCR-21-0410 PMC8530842 33926918

[10] Tie J Wang Y Tomasetti C Li L Springer S Kinde I. Circulating tumor DNA analysis detects minimal residual disease and predicts recurrence in patients with stage II colon cancer. Sci Transl Med. (2016) 8:346ra92. doi: 10.1126/scitranslmed.aaf6219 PMC5346159 27384348

[11] Nakamura Y Tsukada Y Matsuhashi N Murano T Shiozawa M Kato T. Colorectal cancer recurrence prediction using a tissue-free epigenomic minimal residual disease assay. Clin Cancer Res. (2024) 30:2964–73. doi: 10.1158/1078-0432.CCR-24-1651 PMC11443202 39110016

[12] Tsukada Y Matsuhashi N Murano T Shiozawa M Kato T Oki E. Impact of postoperative integrated genomic and epigenomic signatures of circulating tumor DNA (ctDNA) on recurrence in resected colorectal cancer: Initial report of a prospective ctDNA monitoring study COSMOS-CRC-01. JCO. (2022) 40:168. doi: 10.1200/JCO.2022.40.4_suppl.168

[13] Pedersen KS Tan BR Zuo X Kiedrowski LA Bucheit L. Plasma-only multi-omic minimal residual disease (MRD) testing in 2,000 consecutive patients with colorectal cancer (CRC). JCO. (2023) 41:28. doi: 10.1200/JCO.2023.41.4_suppl.28

[14] Grothey A Sobrero AF Shields AF Yoshino T Paul J Taieb J. Duration of adjuvant chemotherapy for stage III colon cancer. N Engl J Med. (2018) 378:1177–88. doi: 10.1056/NEJMoa1713709 PMC6426127 29590544

[15] Gao P Huang X-Z Song Y-X Sun J-X Chen X-W Sun Y. Impact of timing of adjuvant chemotherapy on survival in stage III colon cancer: a population-based study. BMC Cancer. (2018) 18:234. doi: 10.1186/s12885-018-4138-7 29490625 PMC5831576

