# Supplementary figures and images for: Utilization of tissue-free minimal residual disease testing in colorectal cancer patients from Asia and Middle East
Source: Front Oncol. 2024 Sep 20;14:1426941. doi: 10.3389/fonc.2024.1426941 (PMC11449681; doi:10.3389/fonc.2024.1426941)

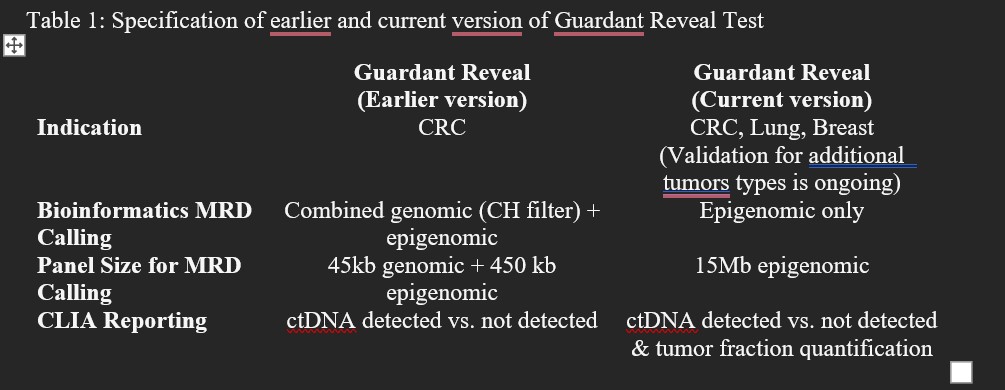

Supplement: Supplementary file 1 [file Image1.jpeg]
